# Magnetoencephalography Reveals a Widespread Increase in Network Connectivity in Idiopathic/Genetic Generalized Epilepsy

**DOI:** 10.1371/journal.pone.0138119

**Published:** 2015-09-14

**Authors:** Adham Elshahabi, Silke Klamer, Ashish Kaul Sahib, Holger Lerche, Christoph Braun, Niels K. Focke

**Affiliations:** 1 Department of Neurology and Epileptology, Hertie Institute for Clinical Brain Research, University of Tübingen, Tübingen, Germany; 2 Werner Reichardt Centre for Integrative Neuroscience, Tübingen, Germany; 3 MEG Center, University of Tübingen, Tübingen, Germany; 4 International Max Planck Research School for Cognitive and Systems Neuroscience, Tübingen, Germany; 5 CIMeC, Center for Mind/Brain Sciences, University of Trento, Trento, Italy; University Paris 6, FRANCE

## Abstract

Idiopathic/genetic generalized epilepsy (IGE/GGE) is characterized by seizures, which start and rapidly engage widely distributed networks, and result in symptoms such as absences, generalized myoclonic and primary generalized tonic-clonic seizures. Although routine magnetic resonance imaging is apparently normal, many studies have reported structural alterations in IGE/GGE patients using diffusion tensor imaging and voxel-based morphometry. Changes have also been reported in functional networks during generalized spike wave discharges. However, network function in the resting-state without epileptiforme discharges has been less well studied. We hypothesize that resting-state networks are more representative of the underlying pathophysiology and abnormal network synchrony. We studied functional network connectivity derived from whole-brain magnetoencephalography recordings in thirteen IGE/GGE and nineteen healthy controls. Using graph theoretical network analysis, we found a widespread increase in connectivity in patients compared to controls. These changes were most pronounced in the motor network, the mesio-frontal and temporal cortex. We did not, however, find any significant difference between the normalized clustering coefficients, indicating preserved gross network architecture. Our findings suggest that increased resting state connectivity could be an important factor for seizure spread and/or generation in IGE/GGE, and could serve as a biomarker for the disease.

## Introduction

Although routine magnetic resonance imaging (MRI) changes are usually unremarkable in idiopathic/genetic generalized epilepsy (IGE/GGE), a growing number of studies are reporting structural alterations in IGE/GGE patients. Diffusion tensor imaging (DTI) studies have shown white matter differences in frontal regions, corpus callosum, thalamo-cortical tracts and the cerebellum [1–5]. Although voxel based morphometry studies have some controversies, it seems that the most consistent alterations are found in the fronto-central regions in juvenile myoclonic epilepsy (JME), and to a lesser extent in IGE/GGE with isolated generalized tonic-clonic seizures (IGE-GTCS) [6–9]. This indicates that there may be some structural “focality” in IGE/GGE. On the functional side, a hallmark in IGE/GGE is generalized spike wave discharges (GSW). Several studies have used electrophysiological and functional imaging modalities to localize the source of these GSW. Thalamus, fronto-mesial regions, insula and the cingulate region have all been reported to be involved, but without a clear understanding of whether these regions may represent the site of seizure origin or are simply part of the propagation [10–13]. Studies investigating functional networks have sought to clarify the role and interplay of brain regions that are involved in IGE/GGE. Increased thalamo-cortical connectivity and deactivation of the default mode network were reported during GSW[14–17]. MEG functional connectivity studies have demonstrated the involvement of the same regions during GSW, mainly the frontal, perinsular and subcortical/thalamic areas [18,19]. On the other hand, Moeller et al. compared fMRI resting state connectivity in intervals containing epileptic activities with discharge-free intervals, and found that the areas involved in GSW did not show abnormal functional connectivity during GSW-free intervals, indicating that different networks may be involved [20]. Similarly, a few studies focused on studying the functional network in GSW-free intervals using MEG and EEG [21–24]. However, the findings were not consistent between these studies. Compared to healthy controls, Clemens et al. found decreased connectivity in low frequencies between 1 and 6 Hz and increased connectivity in higher frequencies [21], while Chavez et al. found increased connectivity in the extended alpha band (5–14 Hz) [23] and Chowdhury et al. found increased connectivity in the lower alpha band (6–9 Hz) [22]. The most recent study by Niso et al. concluded an increased connectivity in all the frequency bands except the alpha band [24]. Most of these studies investigated the network connectivity on the sensor level, i.e. calculating correlations between EEG or MEG sensors limiting direct associations of the results to anatomical brain regions and neural generators.

In this study, we wanted to investigate the IGE/GGE brain networks during the discharge-free intervals using MEG since it provides a superior temporal resolution to fMRI and does not suffer from the referencing problem encountered in EEG. Therefore, it is ideally suited to infer changes of functional connectivity. We opted for a source-space analysis rather than a sensor-space analysis and chose to run a whole-brain analysis rather than a regional analysis since there is limited knowledge about the topology of networks differences in IGE/GGE during GSW-free intervals. We hypothesized that there would be functional network differences between IGE/GGE patients and healthy controls and aimed at identifying the topology of these networks. We postulated that the resting state is better suited to unravel the functional network difference in IGE/GGE since it is not influenced by the strong effects of GSW and may, thus, be more representative of the underlying pathophysiology.

## Methods

This study was conducted in accordance with the Helsinki convention and approved by the local Ethics Committee of the Medical Faculty of the University Tübingen. All participants gave written informed consent prior to participating in the study.

### Participants

The patient group consisted of 18 IGE/GGE patients recruited from the clinic database of the Department of Neurology, University Hospital of Tübingen. Participants with artifacts (e.g. due to excessive movement or noisy measurement) were excluded, leaving 13 patients [9 females; mean (SD) age 38.6 (15.8) years] for the analysis. Out of these 13 patients, five patients were diagnosed with IGE/GGE with isolated generalized tonic-clonic seizures (IGE-GTCS), four patients with childhood absence epilepsy (CAE), two patients with juvenile absence epilepsy (JAE), one patient with juvenile myoclonic epilepsy (JME) and one patient with unspecified idiopathic generalized epilepsy (see S1 Table for clinical data).

Twenty-three healthy controls were initially included in the study. Nineteen participants [11 females; mean (SD) age 38.5 (13.5) years] were available for further analysis. There were no significant age (p = 0.978, t-test) or sex differences (p = 0.515, chi-square) between the groups.

### MEG recording

The recordings were performed using a whole-head 275 channels MEG system (CTF Inc., Vancouver, Canada) in a magnetically shielded room at the MEG center of the University of Tübingen (3906.2 Hz sampling rate). Participants were examined in supine position and were instructed to keep their eyes closed and to not fall asleep. We recorded 15 minutes of spontaneous resting state activity from healthy controls and 30 minutes from IGE/GGE patients to ensure that there would be enough resting state data after excluding epochs with epileptic discharges.

### Data preprocessing

MEG data were visually inspected by an experienced neurologist (S.K.) to identify and mark the GSW. In order to include only resting-state interictal activity, we removed all epochs within a range of ± 10 seconds from the neurologist’s markings. Participants’ data were visually inspected to remove data segments with artifacts (e.g. movement, muscle artifacts) and sensors with noisy signal. Artifact-clean data were segmented into 10-second epochs and downsampled to 409.6 Hz. In total, 30 randomly selected segments were chosen from each participant to be included in further analysis. The segmented data were then filtered with a Butterworth bandpass filter between 0.5 and 70 Hz. Preprocessing and analysis of MEG data was performed using the Fieldtrip toolbox [25] running in Matlab (version 8.3 (R2013b) Mathworks Inc.).

### Anatomical MRI

All participants were scanned using a Siemens MAGNETOM Trio 3T scanner (Siemens AG, Erlangen, Germany), (12-channel array head coil). We acquired a sagittal T1-weighted 3D-MPRAGE sequence as high-resolution anatomical reference (TR 2.3 s, TE 3.03 ms, FA 8°, voxel size 1 x 1 x 1 mm). Structural images for each participant were segmented into tissue classes (skull, scalp, gray matter, white matter, CSF and other) using the unified segmentation toolbox in SPM8 (“new segment”, rev. 3684). The segmented outline of the brain was reconstructed by combining the gray matter, white matter and CSF segments. This outline was used as a volume conduction model for the beamformer source localization of MEG data.

### Spectral analysis

Spectral analysis was performed on the artifact-free sensor level data. To infer the source location of different activity, the cross-spectral density matrix was estimated. We used a multitaper fast Fourier time-frequency transformation with frequency-dependent Discrete Prolate Spheroidal Sequences (DPSS) tapers. The spectral analysis was performed 6 times using different frequencies of interest derived from previous EEG and MEG literature (delta: 2 ± 2 Hz, theta: 6 ± 2 Hz, alpha 10 ± 2 Hz, beta1 16 ± 4 Hz, beta2 25 ± 4 Hz and gamma 40 ± 5 Hz). Spectra from the 6 different frequency bands were selected for further analysis of power and connectivity.

### Source reconstruction

**Fig 1:**
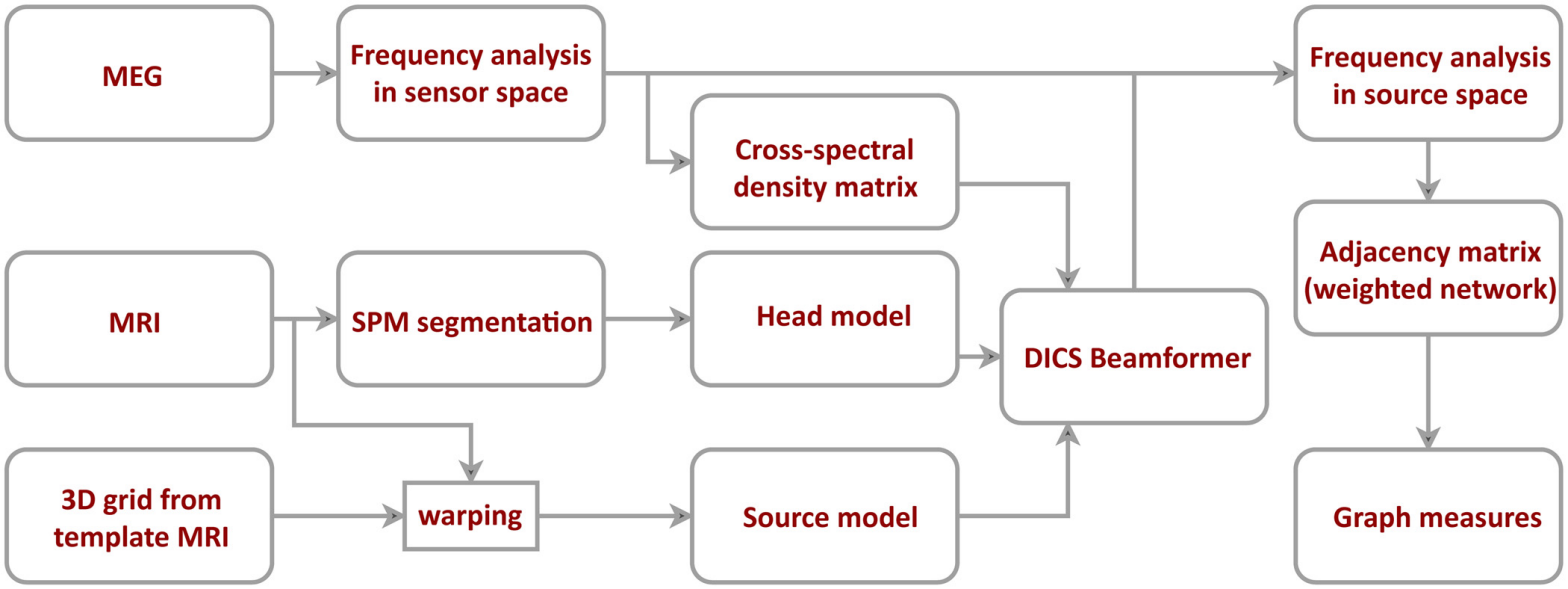
Data processing and network construction pipeline. After artifact rejection, MEG data was projected to the source space using dynamic imaging of coherence sources (DICS) beamformer. The beamformer was based on cross-spectral density matrix and an individual head model. The source model was defined on a regular 3D grid of 1 cm resolution and then warped to fit each participant’s structural MRI. Connectivity between different sources was estimated using the imaginary part of coherence.

To project the data to the source space, we used Dynamic Imaging of Coherent Sources (DICS)—a frequency domain adaptive spatial filtering algorithm [26]. Source positions were based on a regular 3D grid covering the entire brain volume with a resolution of 1 cm. The grid positions were defined on a template MRI in MNI coordinates. We normalized each participant’s MRI image to the template MRI and used the inverse of the resulting transformation matrix to warp the grid positions. Warping the grid approximates each grid position to the same anatomical brain region for each participant. We used only the source positions located in gray matter (SPM grey matter class, probability threshold > 0.5) for further analysis yielding 1015 source positions. For each position, we calculated the leadfield using an individual realistic head model based on participant’s brain shape segmented from MRI. Using both the leadfield and the cross-spectral density matrix of the MEG signal at the frequency of interest, we constructed a spatial filter for each grid position (regularization: lambda = 5%). The spatial filter was calculated for each frequency of interest independently, using the corresponding cross-spectral density matrix for all pairs of channels. These spatial filters were then used to project the Fourier-transformed sensor-level data into the source space. See Fig 1, for an overview on the MEG data analysis pipeline.

### Construction of the connectivity networks

To estimate the connectivity between each pair of source positions, we calculated the coherence between these sources. Coherence is a complex number and its real part reflects the coupling between the signals with 0 time lag. Since this part of coupling is most likely due to volume conduction or a common source, we only used the imaginary part as a true representative of the coupling of two brain regions with a non-zero time lag. See [27] for more technical details. The pairwise calculation of the imaginary coherence resulted in an adjacency matrix between all grey matter sources for each frequency band. We refer to these networks as high-resolution networks. We also analyzed regional connectivity using the automated anatomical labeling (AAL) atlas in MNI space [28]. We averaged all connections between each pair of brain regions (omitting the connections within each brain region) to generate region-to-region connectivity. The resulting networks contained 108 nodes representing 108 distinctive AAL atlas regions. We refer to the resulting networks here as low-resolution networks. These two networks will follow different analysis streams that focus on different aspects of network topology and can yield complementary information.

### Graph measures

The computed connectivity networks were not thresholded and were therefore ‘weighted’ in standard graph theory nomenclature. They were also ‘undirected’ since we used the absolute value of the imaginary part of coherence. We calculated graph theoretical measures directly from these weighted matrices, i.e. the weighted version of the respective [29,30]. For each network, we calculated both global and local measures to assess the characteristics of these networks. We used the nodal strength, clustering coefficient and characteristic path length. Nodal strength of a node in a weighted network is the sum of all its connections with other nodes in the network. The averaged strength of all the nodes in the network is the global nodal strength, and is a measure of overall network connectivity. The clustering coefficient in binary networks is the fraction of connected neighbors of a node. It thus describes the tendency of the node to be involved in a local cluster with neighboring nodes. For weighted networks, the clustering coefficient has been extended by Onnela et al. to be the averaged intensity of the node’s neighbors (sum of all weights between the neighbors) normalized by the unweighted clustering coefficient [30]. The global clustering coefficient is obtained by averaging the clustering coefficient across all nodes and is a measure of network segregation. The length between two nodes is inversely related to the connectivity strength giving lower lengths for stronger connections. Shortest path length between any two nodes in a weighted network is the lowest sum of any possible route between the two nodes either directly or through other nodes. Characteristic path length is the average shortest path length across all nodes in a network. It is a measure of functional integration and how distant regions of the network can communicate with each other. See [29] for a detailed review about these measures and their uses. To normalize the global measures for each network, we generated 100 surrogate networks by randomly shuffling the connections between the nodes. Global graph measures were computed for each surrogate network, and their average over the networks was used to normalize the originally observed global measures [31]. The global and local measures were computed for both high-resolution and low-resolution networks. We also studied the edge weights in low-resolution networks. Graph measures were computed using the Brain Connectivity Toolbox [29].

### Statistical analysis of networks

Group differences of global graph measures were inferred using the nonparametric Mann–Whitney U test. To investigate the topological differences in the high-resolution network, we performed a cluster-based permutation test on the nodal strength values. A first mass univariate t-test was applied to evaluate the between-group difference at each node. This resulted in a t-statistic value per node. We choose a t-statistic threshold of 2.0 and nodes above the threshold were admitted to the next step. Next, the supra-threshold nodes were clustered based on their spatial neighborhood in 3D. To test the significance of these clusters, we ran a Monte Carlo simulation by performing 5000 random permutations where the participants’ networks are shuffled between the two groups. In each permutation, we repeat the process of measuring the t-statistic at each source position, finding supra-threshold positions and identifying clusters. The sum of the t-statistic values of the nodes forming the cluster is calculated and the highest sum from each permutation is used to generate the empirical null distribution. Observed clusters are then tested against the null distribution for significance at p < 0.01 (corrected). To identify regional differences in the low-resolution inter-regional network, we performed a Mann-Whitney U test on the nodal strength of each region between the two groups. We used a p < 0.05 threshold corrected using false discovery rate (FDR) to correct for multiple comparisons [32]. In order to identify the sub-networks that differ between groups, we performed network-based statistics (NBS) on the edges connecting brain regions in the low-resolution networks [33]. NBS follows the same aforementioned cluster-based statistics (initial t-threshold 3.5, 5000 Monte-Carlo permutations, corrected p < 0.05) but with two differences; first, it is performed on the edges rather than on the nodes, second; the cluster is defined as a set of connected edges instead of node neighborhood in physical space. The p-value represents the probability of randomly finding a cluster with the same or greater size under the null hypothesis.

### Power analysis

The power of each of the six different frequency bands of interest was projected into the source space using the DICS filter. For each frequency band, we tested the difference in power between the two groups using non-parametric cluster-based permutation test (initial t-threshold 1.9, 5000 Monte-Carlo permutations, corrected p < 0.05). This approach is identical to the connectivity analysis except a lower p value threshold to capture (potentially) weak effects in power between the two groups.

### Correlation analysis

Finally, we performed a correlation analysis between the connectivity measures and clinical/biometric scores including age and number of years with epilepsy. We used Spearman’s rank correlation coefficient to test the significance of the correlations non-parametrically.

## Results

### Power Analysis

We found significant increase in power in IGE/GGE patients in the delta (p = 0.0336), theta (p = 0.0452) and beta1 (p = 0.0396) bands. The delta band showed a clear bilateral frontal power increase in patients in addition to the right temporal cortex and the cerebellum. Theta band showed a similar topography with more pronounced cerebellar activity. The beta1 band showed more activity in the supplementary motor area in both hemispheres in addition to the regions that are active in the delta and theta bands (S1 Fig).

### Global network characteristics

**Fig 2:**
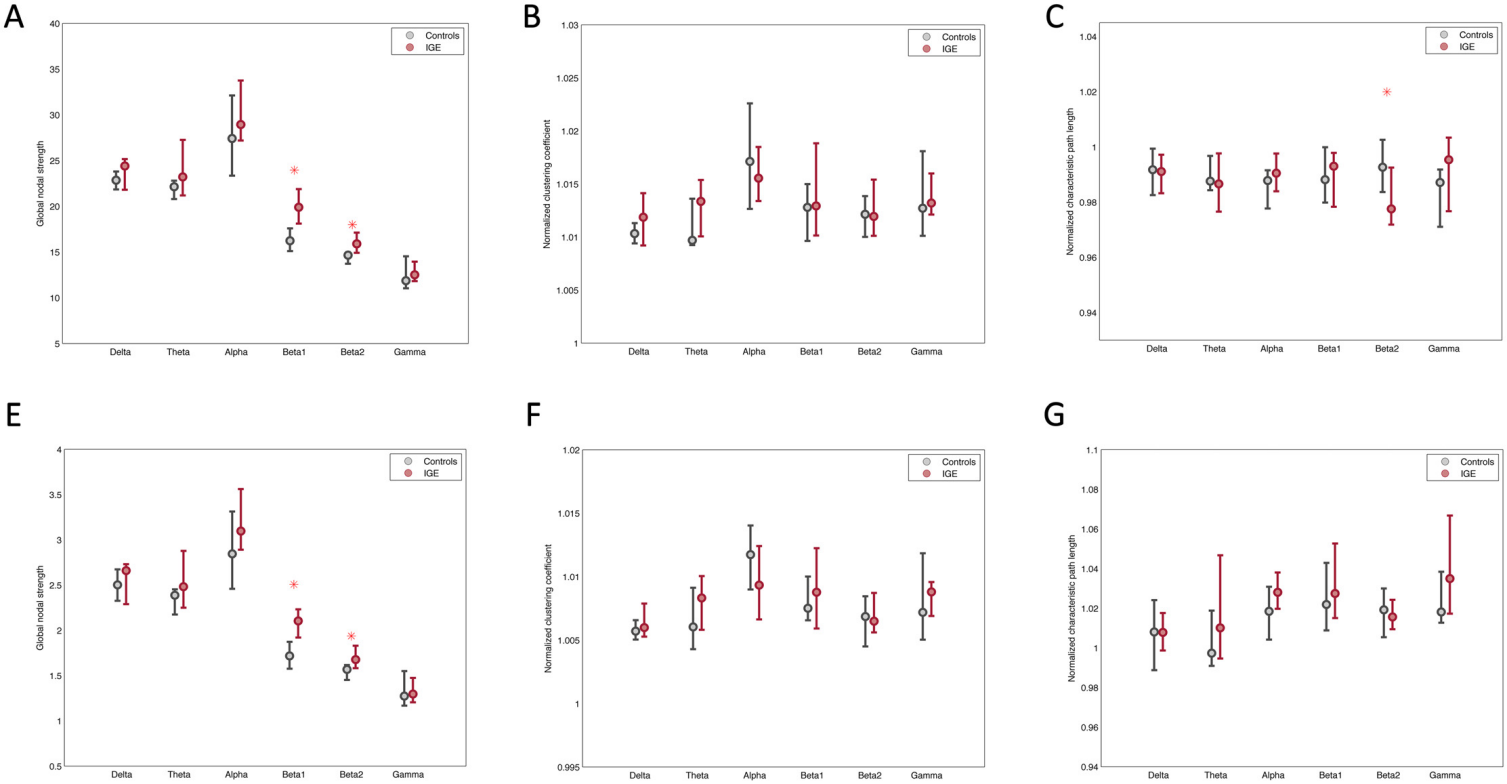
Global network characteristics in high-resolution and low-resolution networks. A) Nodal strength in high-resolution networks B) weighted normalized clustering coefficient in high-resolution networks C) weighted normalized characteristic path length in high-resolution networks D) nodal strength in low-resolution networks E) weighted normalized clustering coefficient in low-resolution networks F) weighted normalized characteristic path length in low-resolution networks. Plots show the medians and the interquartile range across participants in each group. The stars indicate the significant statistical difference between the IGE and healthy control groups using the Mann-Whitney U test. Patients show a significant increase in the nodal strength in both beta1 (p = 0.003) and beta2 (p = 0.0003) bands in high-resolution, as well as in the low-resolution networks (p = 0.005 and p = 0.0003 respectively). Patients also show a significantly lower characteristic path length in the beta2 band in high-resolution network (p = 0.013).

Global nodal strength was significantly increased in IGE/GGE patients in the high-resolution networks in the beta1 (p = 0.003) and beta2 (p = 0.0003) bands as compared to controls. In the low-resolution networks, nodal strength in beta1 and beta2 bands was also significantly higher in patients than in controls (p = 0.005 and p = 0.0003 respectively), and in the alpha band there was a trend for an increased nodal strength in patients (p = 0.066) (compare Fig 2). For the normalized clustering coefficient, there were no significant differences in the low-resolution or high-resolution networks. The normalized characteristic path length in the high-resolution networks showed a significant decrease in IGE/GGE patients in the beta2 band (p = 0.013) (compare Fig 2).

### Local Topological Changes

**Fig 3:**
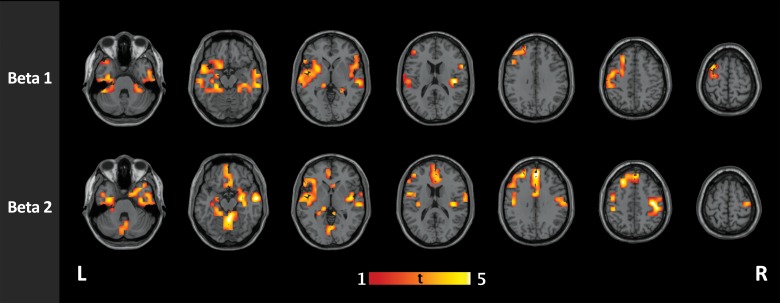
Group comparison of functional connectivity in IGE and healthy controls in high-resolution networks. IGE/GGE patients show clusters of increased connectivity in the beta1 (12–20 Hz) and beta2 (21–29 Hz) bands. In beta1 band, the clusters were mainly located at the left superior temporal gyrus (p = 0.004), the right inferior temporal gyrus (p = 0.011), and the left middle frontal gyrus (p = 0.029). In the beta2 band, four significant clusters were found, located mainly at the left middle frontal gyrus (p = 0.0003), the left fusiform gyrus (p = 0.002), the triangular part of the left inferior frontal guys (p = 0.005) and the right postcentral gyrus (p = 0.034).

Further analysis of nodal strength using cluster-based statistics yielded significant clusters in the beta1 and beta2 bands with higher nodal strength in IGE/GGE patients than in controls. The details of these clusters and their locations are found in Table 1. We found no significant clusters of higher connectivity in controls compared to IGE/GGE patients in any of the frequency bands (Fig 3).

**Table 1:** Clusters resulting from nonparametric between-groups clustering statistics of the nodal strength in high-resolution networks (all grey matter voxels).

**Frequency band**	**Anatomical region (AAL)**	**Cluster size (nodes)**	**Cluster p-value**
Beta1	left superior temporal gyrus	89	0.004
	the right inferior temporal gyrus	54	0.011
	left middle frontal gyrus	29	0.029
Beta2	left middle frontal gyrus	143	0.0003
	left fusiform gyrus	57	0.002
	triangular part of the left inferior frontal gyrus	48	0.005
	right postcentral gyrus	19	0.034

Anatomical brain regions (based on the automated anatomical labeling [AAL] atlas) were determined by the coordinates of the local cluster maximum.

**Fig 4:**
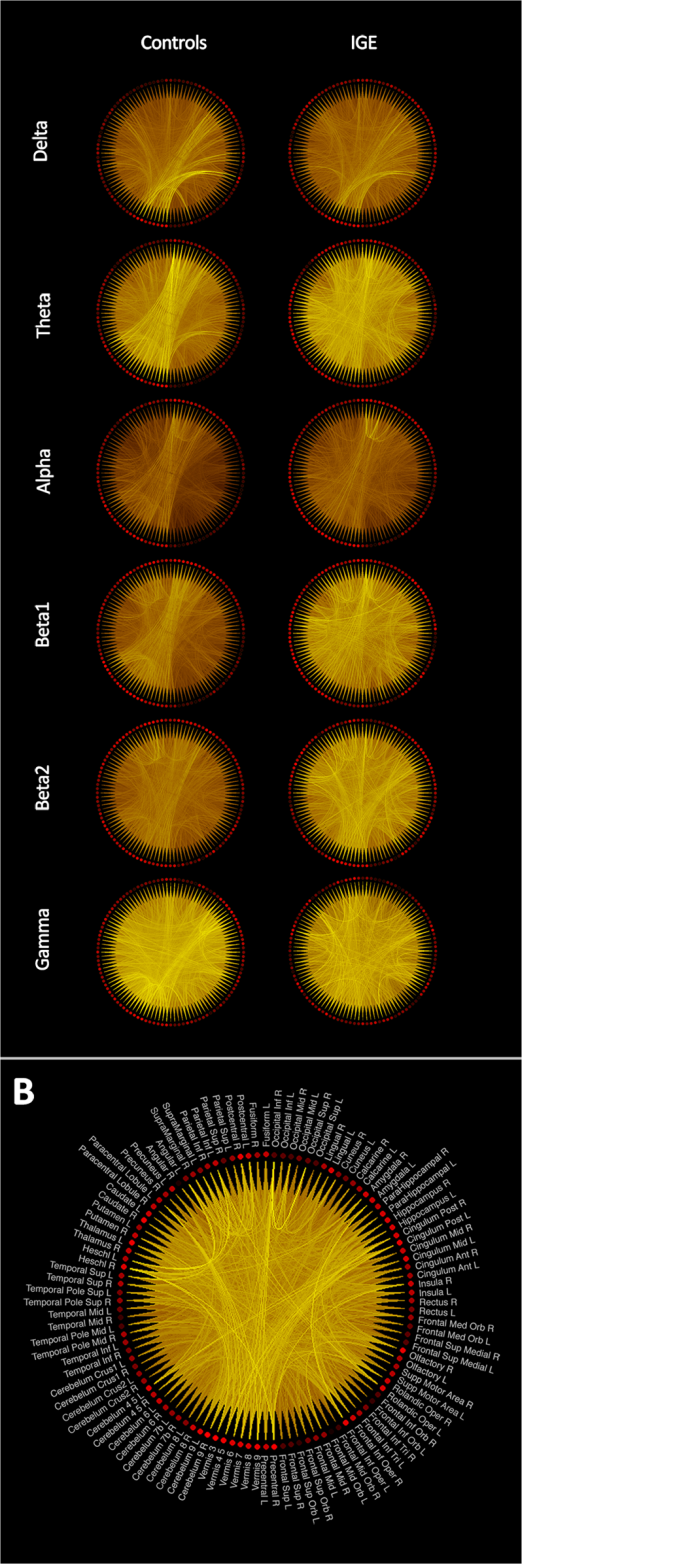
Grand averaged low-resolution connectivity networks across participants of each group. (A) The brightness of the lines connecting regions is proportional to the connectivity value between the two regions. Same color scaling is used for controls and patients’ plots in the same frequency band. (B) The grand average low-resolution network obtained from patients in the beta2 band showing the labels of the AAL regions associated with each node in the network.

The nodal strength in the low-resolution network was significantly higher in IGE/GGE patients than healthy controls in 23 regions for the beta1 band and in 61 regions for the beta2 band. The most significant differences within the beta1 band were found in the precentral and postcentral gyri, the hippocampi and the temporal cortex. Within the beta2 band, most significant differences were found bilaterally in medial superior frontal gyrus, amygdala, thalamus and the cerebellum. Other frequency bands did not show any significant regional difference after FDR correction and we did not find any region with higher nodal strength in healthy subjects than IGE/GGE patients. The complete list of regions and their statistical values is presented in S2 Table and a rendering of grand average networks is shown in Fig 4.

**Fig 5:**
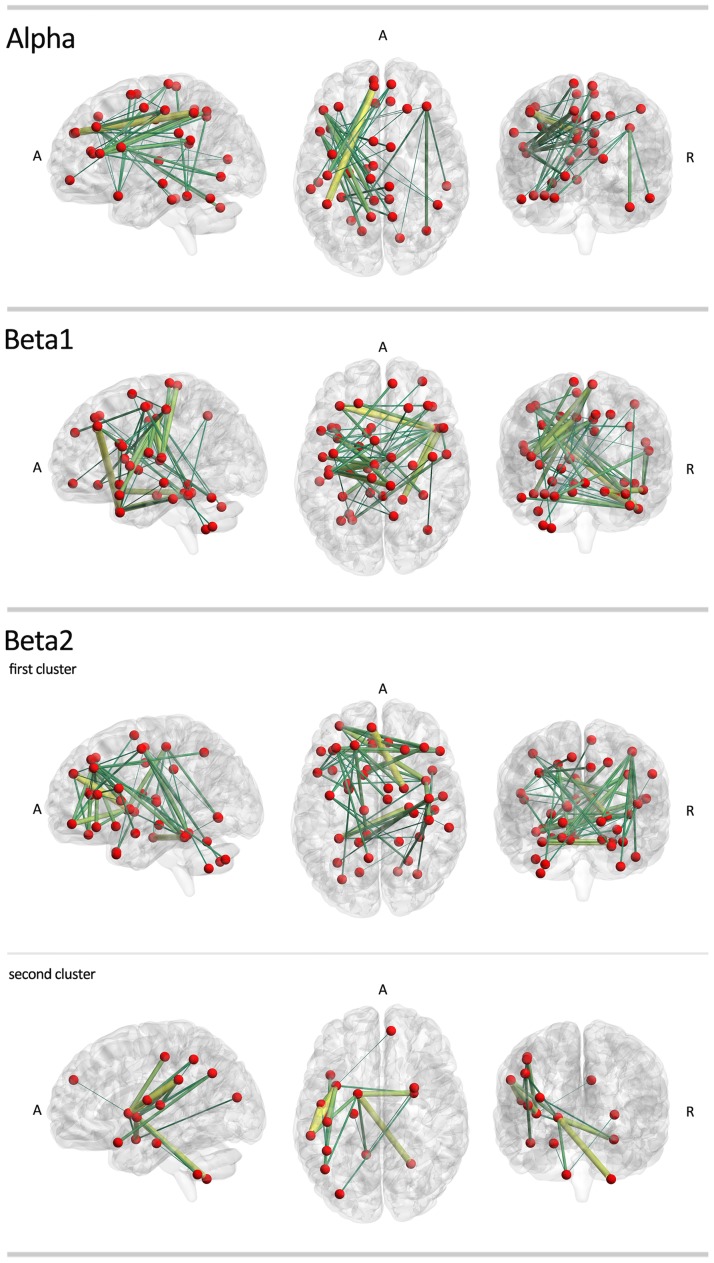
Group comparison of functional connectivity in IGE/GGE and healthy controls in low-resolution networks. IGE/GGE patients show sub-networks of edges with significantly higher connectivity values in the alpha, beta1 and beta2 bands. Note the particular involvement of motor-networks. No significant clusters were found with higher connectivity in healthy controls. Color and size codes are proportional to the t-statistic value. The brain networks were visualized with the BrainNet Viewer [34].

Using network-based statistics, we found significant sub-networks with higher connectivity in IGE/GGE patients. The differences were found in alpha, beta1 and beta2 bands. In the alpha band, a significant extensive cluster included edges connecting the frontal, parietal cortex and cerebellum (p = 0.013). The most significant connections in this sub-network were between the left superior frontal gyrus and the right inferior parietal gyrus (t = 6.702), as well as between the left inferior frontal gyrus and the left precuneus (t = 5.196). In the beta1 band, we found another extensive network with increased connectivity in IGE/GGE patients (p = 0.003). The most significant connections were between the left middle frontal gyrus and the right inferior temporal gyrus (t = 5.170), and between the right middle temporal gyrus and the left hemispheric lobule of the cerebellum (t = 4.820). In the beta2 band, two clusters were found. One extensive cluster covered mainly the frontal cortex (p = 0.0008) with the most significant edges between the medial part of the superior frontal gyrus and the right thalamus (t = 4.965), right parahippocampus and left fusiform gyrus (t = 4.710), and between the left middle frontal gyrus and the right insula (t = 4.505). A smaller cluster showed a significant increase in connectivity in IGE/GGE patients (p = 0.012), mainly between the left insula and left supramarginal gyrus (t = 4.625), and between the left supramarginal gyrus and left rolandic operculum (t = 4.538). We did not find any significant clusters of decreased connectivity in IGE/GGE patients (see Fig 5 and S3 Table).

### Correlation analysis

We correlated global nodal strength with age for all participants, and number of years with epilepsy for IGE/GGE patients. A correlation was performed on the frequency bands that showed a significant difference between the patients and controls. We did not find any significant correlation between the nodal strength and age (beta1: p = 0.817, R = -0.043, beta2: p = 0.952, R = 0.011). There was also no significant correlation between the nodal strength and the number of years with epilepsy in patients (beta1: p = 0.272, R = 0.330, beta2: p = 0.404, R = -0.253).

## Discussion

We found significant functional resting-state network differences between IGE/GGE patients and controls using a whole brain analysis. The most striking finding was a general increase in connectivity in IGE/GGE patients. This was evident in the global network statistics as well as in the edge-wise and low-resolution regional analyses. Increased connectivity is a biologically plausible mechanism to explain the generation, or more rapid spread, of spontaneous seizures. Interestingly, the normalized clustering coefficient was not significantly different between patients and controls and the normalized characteristic path length showed only a marginal difference, indicating that the gross network topology is preserved. This is also evident in Fig 4, which shows increased connectivity but similar topology. It is important to highlight that we took great care to analyze only interictal periods without GSW. During seizures or GSW, that may represent subclinical seizures, different mechanisms and other structures could be involved, leading to possibly different patterns in functional connectivity and network topology.

Even with globally increased connectivity some areas and connections were preferentially affected. Using a clustering strategy, both on edges as well as nodes, we could demonstrate a particular involvement of frontal and temporal regions and the motor network, both in the high-resolution as well as the low-resolution network analysis. We observed the most significant increase in connectivity in the superior frontal gyrus, precentral and postcentral gyri, temporal cortex and the cerebellum. These results are similar to the increased connectivity in the sensory-motor and supplementary motor cortex found in JME patients using fMRI and DTI [35,36] indicating that these regions might not be exclusive for JME but rather involved in IGE/GGE syndromes in general given the low prevalence of JME in our cohort. In structural imaging studies, DTI alterations with a focus on the mesio-frontal area have also been shown [1,3–5,37]. Moreover, we found a strong involvement of the cerebellum. Considering its importance for motor functions, it is not surprising to find the cerebellum as part of the increased motor connectivity. Structural differences in DTI of the cerebellum were reported in IGE/GGE patients as well [3]. However, it is not clear whether these differences are merely an indirect consequence of motor system alterations, or have an independent contribution to IGE/GGE. We also found significantly increased regional connectivity in the temporal lobes (superior and inferior temporal gyri) and Insula, again indicating that this phenomenon is not limited to frontal and motor networks.

There has been some debate as to whether the seemingly focal changes in JME, and potentially IGE/GGE in general, are truly focal or merely “the tip of the iceberg” [38]. Our results seem to support the latter interpretation. Although the frontal lobe may be preferentially involved, the underlying process is global, which is plausible with regards to a genetically defined disease. This is in line with our findings in a different IGE/GGE cohort that also showed widespread structural DTI differences, again with a frontal focus, but clearly going beyond that [2]. Previous studies using a spatially restricted approach, or functional tasks that activate only limited areas of the brain, could underestimate the true spatial extent [5,35,39].

There is a long-standing and unsolved debate if IGE/GGE sub-syndromes are biologically separate entities even if their clinical symptomatology is distinct. It has been shown that 15q13.3 microdeletions, one of the most prevalent genetic risk-factors for IGE/GGE, are present in various IGE/GGE sub-syndromes [40]. Even in monogenic, familial epilepsies (e.g. *SCN1A* loss-of-function), there is a strong heterogeneity of the clinical phenotype [41]. In our study we intentionally did not restrict the analysis to specific IGE/GGE sub-syndromes but rather aimed to find a potential common mechanism. Our findings indicate that increased network connectivity may be such a common mechanism in IGE/GGE and not a hallmark of sub-syndromes like JME. From our data, we cannot infer if the overall functional connectivity or the regional focality would be different in more selected cohorts and specific IGE/GGE sub-syndromes. However, in a previous study we found no significant structural differences between patients with JME and IGE/GGE, and both showed very similar differences compared to controls [2]. Although this was a completely different cohort, the spatial overlap of the functional and structural differences is noteworthy [2]. It seems that there is a pattern of increased functional connectivity between regions that exhibit a decrease in the white matter anisotropy. Although this pattern is counter-intuitive at first, it has been described by others [42], indicating that this may be part of a common mechanism. A possible explanation could be the loss, or architectural reduction, of long-reaching inhibitory fibers, compatible with both a reduction of anisotropy and yet increased functional connectivity.

It is also interesting that increases in thalamic connectivity were relatively limited compared to the strong differences found in the frontal and parietal regions. This may seem surprising since this is the most consistent finding in the EEG-fMRI studies of GSW [10,11,13,15]. However, it is likely that increased connectivity of the thalamus is an important factor of GSW but not necessary by an architectural difference in IGE/GGE per se. Also, MEG may not be ideally suited to study deep sources like the thalamus. The global increase in connectivity, however, challenges the notion found in ROI-based studies that the IGE/GGE brain functions normally during GSW-free intervals [20].

In our cohort the most significant differences were found in the beta band. Beta band was reported previously to have increased connectivity in several MEG and EEG studies in IGE/GGE, as well as temporal and frontal lobe epilepsies [24,43,44]. This may—in part—be related to the fact that beta is the dominant frequency domain of motor and frontal networks [45]. Many EEG and MEG studies on various epilepsy sub-syndromes found network changes to be also in the theta band [21,46]. We found power differences between the groups in the beta, delta and theta bands. The delta and theta bands did not show significant connectivity alterations indicating that the power differences do not necessarily reflect the connectivity patterns. In addition, since the imaginary part of coherence only reflects phase-shifted connectivity, differences in power should have limited effects on the connectivity differences between the two groups [27].

### Technical considerations

The study of functional connectivity using electrophysiological data is currently challenging due to a number of technical factors. One factor is the connectivity measure used. Different measures are based on different theoretical concepts on how two brain regions are functionally connected [27,47]. Although the performance of many of these measures is to some degree similar, different measures capture different aspects of the relationship between two signals. We chose the imaginary part of coherence because it minimizes the effect of the volume conduction, which is an important source of false connectivity and cannot be avoided even with high-density MEG, individual MRI-based head-models and state-of-the-art source reconstruction methods. Signal power is another important factor that can alter interpreting connectivity differences between groups. However, by using the imaginary part of coherence, we look into the time-shifted coherence between different regions, which has very limited influence of power [27].

### Limitations

Our study was, after vigorous data inspection and exclusion, based on a relatively small sample size of 32 participants (13 patients and 19 controls). Nonetheless, we found significant network differences even after correcting for multiple comparisons needed in full-brain analysis. Thus, our study was adequately powered to answer the question of the presence of network changes in IGE/GGE in the resting state. We chose not to correct for testing in multiple frequency bands since we did not have a clear hypothesis of interplay between the frequency bands in this study. However, most of our findings are highly significant and would survive a Bonferroni correction against the factor of 12 (6 frequency bands and 2 analysis streams). In addition, twelve out of the thirteen studied patients were on anti-epileptic medication. Thus, in our cohort, drug effects may have contributed to the results. However, a previous study has found connectivity differences between controls and drug-naïve IGE/GGE patients using EEG [21]. Moreover, two recent studies have compared connectivity in healthy controls with IGE/GGE patients and also with their unaffected, non-medicated siblings [22,39]. These two studies used two different modalities, fMRI and EEG, and concluded that the connectivity differences observed between the patients and controls were also observed between the controls and the unaffected siblings suggesting that the connectivity differences are not solely due to anti-epileptic drugs. Network imaging by means of graph theory is a novel method that is not yet extensively validated. Using different approaches (voxel-based versus regional networks and edge-based versus node-based statistics) we found very similar results. This is re-assuring since it shows that these methodological choices do not alter the biological interpretation. Finally, we cannot exclude that even more widespread alterations exist that could be detected by a larger group. If specific differences of functional connectivity exists between clinically distinct IGE/GGE sub-syndromes has to be assessed in further studies.

### Conclusions

In summary, we showed that IGE/GGE patients, compared to controls, have widespread increased functional network connectivity in the resting state, with a particular focus on the motor network but clearly extending beyond this. The increased connectivity is present in GSW-free intervals, and could be related to an increased likelihood to generate spontaneous, generalized seizures in these patients.

## Supporting Information

S1 Fig Significant power clusters between the two groups. The panels show the t-maps of the statistical difference and are masked to show only significant positions. Higher values refer to higher power in patients than in healthy controls. The three panels show the t-maps of the following frequency bands: A) Delta band (0.5–4 Hz). B) Theta band (4–8 Hz). C) Beta1 band (12–20 Hz).
(TIF)

S1 Table Clinical data of IGE/GGE patients. CAE = childhood absence epilepsy; JAE = juvenile absence epilepsy; JME = juvenile myoclonic epilepsy; GTCS = generalized tonic-clonic seizure only. CLB = clobazam; ESL = eslicarbazepine; LEV = levetiracetam; LTG = lamotrigine; TPM = topiramate; VPA = valproate.
(DOCX)

S2 Table Brain regions with a significant increase in connectivity (nodal strength) in IGE/GGE patients compared to healthy controls. Statistics based on Mann-Whitney U test on connectivity of each region between patients and healthy controls (FDR corrected). The regions list is sorted based on the corrected-p value then on the uncorrected p-value.
(DOCX)

S3 Table Most significant inter-regional connections resulting from edge-wise network based statistics. Four clusters of edges were found in three frequency bands (alpha, beta1 and beta2) with higher connections in patients than in healthy controls. The table lists the top 10 connections in each cluster based on the t-value. There were no clusters with higher connectivity in controls than in patients.
(DOCX)
