# Supplementary figures and images for: Magnetoencephalography Reveals a Widespread Increase in Network Connectivity in Idiopathic/Genetic Generalized Epilepsy
Source: PLoS One. 2015 Sep 14;10(9):e0138119. doi: 10.1371/journal.pone.0138119 (PMC4569354; doi:10.1371/journal.pone.0138119)

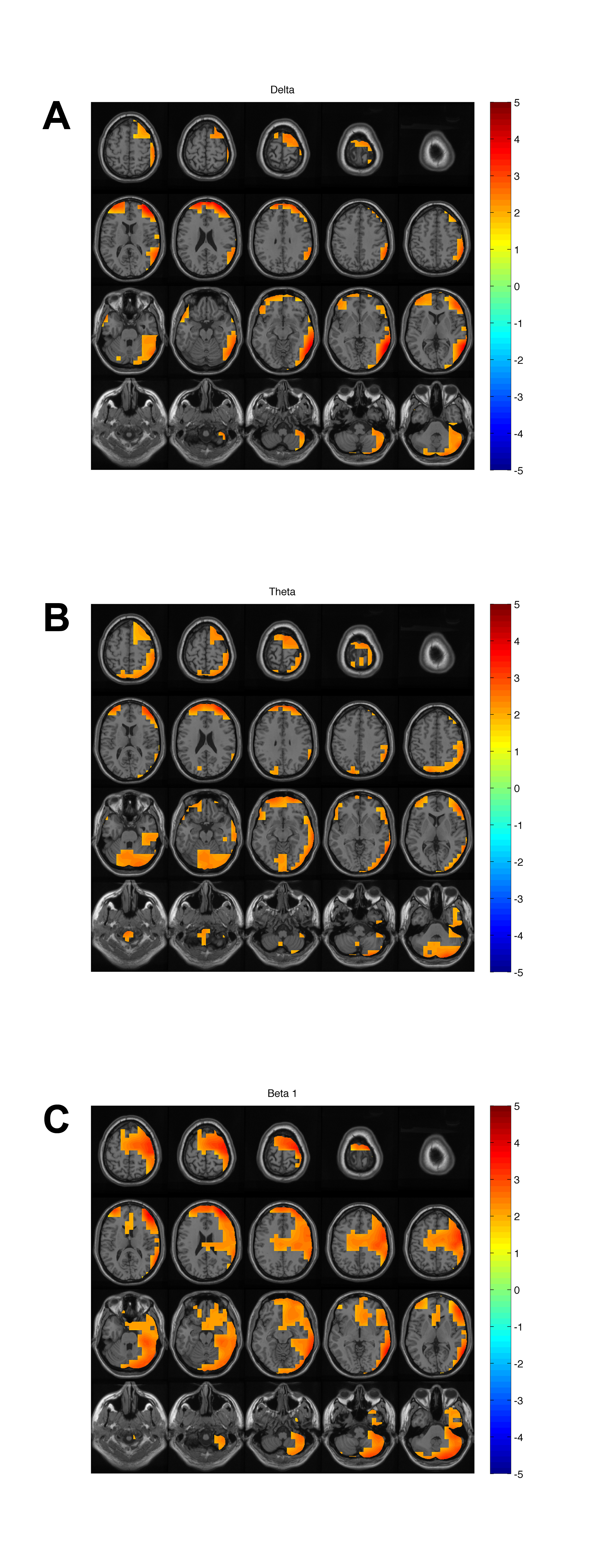

Supplement: S1 Fig — The panels show the t-maps of the statistical difference and are masked to show only significant positions. Higher values refer to higher power in patients than in healthy controls. The three panels show the t-maps of the following frequency bands: A) Delta band (0.5–4 Hz). B) Theta band (4–8 Hz). C) Beta1 band (12–20 Hz). (TIF) [file pone.0138119.s001.tif]
