# Supplementary material for: Magnetoencephalography Reveals a Widespread Increase in Network Connectivity in Idiopathic/Genetic Generalized Epilepsy
Source: PLoS One. 2015 Sep 14;10(9):e0138119. doi: 10.1371/journal.pone.0138119 (PMC4569354; doi:10.1371/journal.pone.0138119)
Supplement: S1 Table — CAE = childhood absence epilepsy; JAE = juvenile absence epilepsy; JME = juvenile myoclonic epilepsy; GTCS = generalized tonic-clonic seizure only. CLB = clobazam; ESL = eslicarbazepine; LEV = levetiracetam; LTG = lamotrigine; TPM = topiramate; VPA = valproate. (DOCX) [file pone.0138119.s002.docx]

| Patient | Age/Onset | Diagnosis | Spikes/30 min | AED | Dose |
| --- | --- | --- | --- | --- | --- |
| 1 | 43/12 | IGE[GTCS] | 64 | ESL, LEV | 2000 mg and 2000 mg |
| 2 | 58/unclear | IGE | 208 | LTG, TPM | 400 mg and 400 mg |
| 3 | 21/4 | CAE | 0 | LEV | 1500 mg |
| 4 | 22/11 | JAE | 0 | LTG | 225 mg |
| 5 | 31/15 | JAE | 0 | VPA, LEV | 2000 mg and 1000 mg |
| 6 | 71/13 | IGE[GTCS] | 620 | TPM | 200 mg |
| 7 | 19/6 | CAE | 0 | LTG | 250 mg |
| 8 | 50/48 | IGE[GTCS] | 0 | LEV | 1000 mg |
| 9 | 47/38 | IGE[GTCS] | 0 | LEV, CLB | 2000 mg and 2.5 mg |
| 10 | 47/10 | JME | 0 | LTG | 100 mg |
| 11 | 32/7 | CAE | 0 | LEV | 1000 mg |
| 12 | 24/6 | CAE | 21 | none | - |
| 13 | 37/16 | IGE[GTCS] | 0 | LEV | 2000 mg |
