# Supplementary material for: Magnetoencephalography Reveals a Widespread Increase in Network Connectivity in Idiopathic/Genetic Generalized Epilepsy
Source: PLoS One. 2015 Sep 14;10(9):e0138119. doi: 10.1371/journal.pone.0138119 (PMC4569354; doi:10.1371/journal.pone.0138119)
Supplement: S2 Table — Statistics based on Mann-Whitney U test on connectivity of each region between patients and healthy controls (FDR corrected). The regions list is sorted based on the corrected-p value then on the uncorrected p-value. (DOCX) [file pone.0138119.s003.docx]

| Frequency | AAL region | U(32) | p | p (FDR corrected) |
| --- | --- | --- | --- | --- |
| Beta1 | Frontal_Sup_L | 222 | 0.0002 | 0.012 |
|  | Thalamus_L | 219 | 0.0003 | 0.012 |
|  | Frontal_Inf_Tri_L | 216 | 0.0004 | 0.012 |
|  | Caudate_L | 213 | 0.0006 | 0.012 |
|  | Amygdala_R | 214 | 0.0006 | 0.012 |
|  | Frontal_Sup_Medial_L | 211 | 0.0008 | 0.012 |
|  | Frontal_Sup_Medial_R | 211 | 0.0008 | 0.012 |
|  | Hippocampus_R | 207 | 0.001 | 0.012 |
|  | Postcentral_R | 207 | 0.001 | 0.012 |
|  | Frontal_Inf_Oper_L | 208 | 0.001 | 0.012 |
|  | Vermis_4_5 | 208 | 0.001 | 0.012 |
|  | Cingulum_Post_L | 209 | 0.001 | 0.012 |
|  | Insula_R | 210 | 0.001 | 0.012 |
|  | Precentral_L | 207 | 0.001 | 0.037 |
|  | Rolandic_Oper_R | 203 | 0.002 | 0.037 |
|  | Insula_L | 203 | 0.002 | 0.037 |
|  | Hippocampus_R | 204 | 0.002 | 0.037 |
|  | SupraMarginal_R | 204 | 0.002 | 0.037 |
|  | Postcentral_R | 205 | 0.002 | 0.037 |
|  | Temporal_Mid_R | 206 | 0.002 | 0.037 |
|  | ParaHippocampal_R | 201 | 0.003 | 0.037 |
|  | ParaHippocampal_L | 197 | 0.005 | 0.037 |
|  | Temporal_Inf_R | 197 | 0.005 | 0.037 |
|  | Cingulum_Mid_R | 198 | 0.005 | 0.037 |
|  | Amygdala_R | 198 | 0.005 | 0.037 |
|  | Frontal_Mid_L | 195 | 0.006 | 0.037 |
|  | Cingulum_Mid_L | 195 | 0.006 | 0.037 |
|  | Hippocampus_L | 195 | 0.006 | 0.037 |
|  | Putamen_L | 195 | 0.006 | 0.037 |
|  | Temporal_Sup_L | 195 | 0.006 | 0.037 |
|  | Temporal_Sup_R | 195 | 0.006 | 0.037 |
|  | Cerebelum_4_5_R | 196 | 0.006 | 0.037 |
|  | Postcentral_L | 194 | 0.007 | 0.037 |
|  | Temporal_Pole_Sup_R | 194 | 0.007 | 0.037 |
|  | Temporal_Pole_Sup_L | 192 | 0.009 | 0.045 |
|  | Rolandic_Oper_L | 191 | 0.010 | 0.048 |
| Beta2 | Precentral_L | 203 | 0.002 | 0.012 |
|  | Precentral_R | 206 | 0.002 | 0.012 |
|  | Rolandic_Oper_L | 204 | 0.002 | 0.012 |
|  | Insula_L | 203 | 0.002 | 0.012 |
|  | Cingulum_Ant_L | 204 | 0.002 | 0.012 |
|  | ParaHippocampal_R | 205 | 0.002 | 0.012 |
|  | Lingual_L | 204 | 0.002 | 0.012 |
|  | Caudate_R | 204 | 0.002 | 0.012 |
|  | Vermis_6 | 206 | 0.002 | 0.012 |
|  | Parietal_Inf_R | 202 | 0.003 | 0.012 |
|  | Temporal_Sup_L | 202 | 0.003 | 0.012 |
|  | Frontal_Mid_L | 201 | 0.003 | 0.013 |
|  | Postcentral_L | 201 | 0.003 | 0.013 |
|  | Temporal_Sup_R | 201 | 0.003 | 0.013 |
|  | Fusiform_L | 199 | 0.004 | 0.013 |
|  | Fusiform_R | 199 | 0.004 | 0.013 |
|  | Putamen_R | 200 | 0.004 | 0.013 |
|  | Thalamus_R | 199 | 0.004 | 0.013 |
|  | Cerebelum_4_5_L | 200 | 0.004 | 0.013 |
|  | Rolandic_Oper_R | 200 | 0.006 | 0.013 |
|  | Hippocampus_L | 198 | 0.005 | 0.014 |
|  | Cingulum_Mid_L | 197 | 0.005 | 0.015 |
|  | Vermis_3 | 197 | 0.005 | 0.015 |
|  | Cingulum_Post_R | 196 | 0.006 | 0.016 |
|  | Temporal_Pole_Mid_R | 196 | 0.006 | 0.016 |
|  | Temporal_Inf_R | 196 | 0.006 | 0.016 |
|  | Frontal_Mid_R | 194 | 0.007 | 0.019 |
|  | Cingulum_Ant_R | 194 | 0.007 | 0.019 |
|  | ParaHippocampal_L | 194 | 0.007 | 0.019 |
|  | Rectus_L | 193 | 0.008 | 0.020 |
|  | Olfactory_R | 192 | 0.009 | 0.022 |
|  | Vermis_9 | 192 | 0.009 | 0.022 |
|  | Cerebelum_8_R | 191 | 0.010 | 0.023 |
|  | Cerebelum_9_R | 191 | 0.010 | 0.023 |
|  | SupraMarginal_R | 190 | 0.011 | 0.025 |
|  | Vermis_8 | 190 | 0.011 | 0.025 |
|  | Vermis_7 | 189 | 0.013 | 0.027 |
|  | Cingulum_Mid_R | 188 | 0.014 | 0.030 |
|  | Temporal_Inf_L | 187 | 0.016 | 0.033 |
|  | Frontal_Mid_Orb_L | 186 | 0.017 | 0.035 |
|  | Temporal_Pole_Sup_R | 186 | 0.017 | 0.035 |
|  | Frontal_Med_Orb_L | 185 | 0.019 | 0.037 |
|  | Lingual_R | 185 | 0.019 | 0.037 |
|  | Parietal_Inf_L | 184 | 0.021 | 0.040 |
|  | Heschl_L | 184 | 0.021 | 0.040 |
|  | Amygdala_L | 183 | 0.024 | 0.043 |
|  | Cerebelum_6_R | 182 | 0.026 | 0.046 |
|  | Cerebelum_7b_R | 182 | 0.026 | 0.046 |
