# Supplementary material for: Magnetoencephalography Reveals a Widespread Increase in Network Connectivity in Idiopathic/Genetic Generalized Epilepsy
Source: PLoS One. 2015 Sep 14;10(9):e0138119. doi: 10.1371/journal.pone.0138119 (PMC4569354; doi:10.1371/journal.pone.0138119)
Supplement: S3 Table — Four clusters of edges were found in three frequency bands (alpha, beta1 and beta2) with higher connections in patients than in healthy controls. The table lists the top 10 connections in each cluster based on the t-value. There were no clusters with higher connectivity in controls than in patients. (DOCX) [file pone.0138119.s004.docx]

| Frequency Band | AAL region 1 | AAL region 2 | t-value |
| --- | --- | --- | --- |
| Alpha | Frontal_Sup_Medial_L | Parietal_Inf_L | 6.702 |
|  | Frontal_Inf_Tri_L | Precuneus_L | 5.196 |
|  | Frontal_Inf_Tri_L | Cingulum_Post_L | 4.920 |
|  | Frontal_Sup_Medial_L | Postcentral_L | 4.816 |
|  | Frontal_Mid_R | Cerebelum_Crus2_R | 4.747 |
|  | Frontal_Inf_Oper_L | Precuneus_L | 4.698 |
|  | Frontal_Inf_Tri_L | Cingulum_Mid_L | 4.518 |
|  | Frontal_Inf_Oper_L | Lingual_L | 4.441 |
|  | Cingulum_Ant_L | Parietal_Inf_L | 4.412 |
|  | Frontal_Inf_Tri_L | Paracentral_Lobule_L | 4.396 |
| Beta1 | Frontal_Mid_L | Temporal_Inf_R | 5.170 |
|  | Temporal_Mid_R | Cerebelum_7b_L | 4.820 |
|  | Temporal_Inf_R | Cerebelum_7b_L | 4.785 |
|  | Cingulum_Mid_L | Temporal_Sup_L | 4.756 |
|  | Paracentral_Lobule_R | Temporal_Sup_L | 4.599 |
|  | Insula_L | Cingulum_Mid_L | 4.523 |
|  | Paracentral_Lobule_R | Temporal_Mid_L | 4.392 |
|  | Fusiform_L | Temporal_Inf_R | 4.314 |
|  | Rolandic_Oper_L | Cingulum_Mid_L | 4.302 |
|  | Cingulum_Mid_R | Temporal_Sup_L | 4.301 |
| Beta2 (first cluster) | Frontal_Sup_Medial_L | Thalamus_R | 4.965 |
|  | ParaHippocampal_R | Fusiform_L | 4.710 |
|  | Frontal_Mid_Orb_L | Insula_R | 4.505 |
|  | Insula_R | Postcentral_R | 4.297 |
|  | Frontal_Inf_Tri_R | Frontal_Inf_Orb_L | 4.214 |
|  | Frontal_Mid_R | Olfactory_L | 4.203 |
|  | Frontal_Mid_Orb_L | Frontal_Inf_Tri_R | 4.200 |
|  | Precentral_R | Cerebelum_6_R | 4.199 |
|  | Rectus_L | Cingulum_Ant_L | 4.167 |
|  | Frontal_Sup_L | Cerebelum_6_L | 4.126 |
| Beta2 (second cluster) | Insula_L | SupraMarginal_L | 4.625 |
|  | Rolandic_Oper_L | SupraMarginal_L | 4.538 |
|  | Thalamus_L | Vermis_3 | 4.491 |
|  | Amygdala_R | Thalamus_L | 4.340 |
|  | Insula_L | Postcentral_L | 4.176 |
|  | Rolandic_Oper_L | Insula_L | 4.077 |
|  | SupraMarginal_L | Thalamus_L | 3.982 |
|  | SupraMarginal_L | Temporal_Sup_L | 3.954 |
|  | Angular_L | Temporal_Mid_L | 3.931 |
|  | Insula_L | Temporal_Sup_L | 3.902 |
